# First Detection of an Enterovirus C99 in a Captive Chimpanzee with Acute Flaccid Paralysis, from the Tchimpounga Chimpanzee Rehabilitation Center, Republic of Congo

**DOI:** 10.1371/journal.pone.0136700

**Published:** 2015-08-24

**Authors:** Illich Manfred Mombo, Nicolas Berthet, Alexander N. Lukashev, Tobias Bleicker, Sebastian Brünink, Lucas Léger, Rebeca Atencia, Debby Cox, Christiane Bouchier, Patrick Durand, Céline Arnathau, Lionel Brazier, Joseph N. Fair, Bradley S. Schneider, Jan Felix Drexler, Franck Prugnolle, Christian Drosten, François Renaud, Eric M. Leroy, Virginie Rougeron

**Affiliations:** 1 Centre International de Recherche Médicale de Franceville, BP769, Franceville, Gabon; 2 Laboratoire MIVEGEC UMR 224–5290 CNRS-IRD-UM1-UM2, IRD, Montpellier, France; 3 Centre National de la Recherche Scientifique, UMR3569, 25 rue du docteur Roux, 75724, Paris, France; 4 Chumakov Institute of Poliomyelitis and Viral Encephalities, Moscow, Russia; 5 Institute of Virology, University of Bonn Medical Centre, Bonn, Germany; 6 The Jane Goodall Institute, Suite 550, 1595 Spring Hill Rd, Vienna, Virginia, 22182, United States of America; 7 Institut Pasteur, Genomic platform, 28, rue du Docteur Roux, F-75724, Paris, France; 8 Metabiota, Inc., 1 Sutter Street, Suite 600, San Francisco, California, 94104, United States of America; 9 CHRU de Montpellier, Montpellier, France; The Scripps Research Institute, UNITED STATES

## Abstract

Enteroviruses, members of the *Picornaviridae* family, are ubiquitous viruses responsible for mild to severe infections in human populations around the world. In 2010 Pointe-Noire, Republic of Congo recorded an outbreak of acute flaccid paralysis (AFP) in the humans, caused by wild poliovirus type 1 (WPV1). One month later, in the Tchimpounga sanctuary near Pointe-Noire, a chimpanzee developed signs similar to AFP, with paralysis of the lower limbs. In the present work, we sought to identify the pathogen, including viral and bacterial agents, responsible for this illness. In order to identify the causative agent, we evaluated a fecal specimen by PCR and sequencing. A Human enterovirus *C*, specifically of the EV-C99 type was potentially responsible for the illness in this chimpanzee. To rule out other possible causative agents, we also investigated the bacteriome and the virome using next generation sequencing. The majority of bacterial reads obtained belonged to commensal bacteria (95%), and the mammalian virus reads matched mainly with viruses of the *Picornaviridae* family (99%), in which enteroviruses were the most abundant (99.6%). This study thus reports the first identification of a chimpanzee presenting AFP most likely caused by an enterovirus and demonstrates once again the cross-species transmission of a human pathogen to an ape.

## Introduction

In the last few years, many pathogens capable of infecting humans, such as Ebola virus [1, 2], simian immunodeficiency viruses [3], respiratory viruses [4, 5], anthrax [6], herpes viruses [7] as well as enteroviruses [8, 9] have been identified in great apes. Contacts between human populations and great apes as well as monkeys are frequent and increasing, due to commercial poaching, deforestation, and monitoring [10–13]. The direct consequence is the increased likelihood of pathogens exchanges and thus of emerging zoonoses and antropozoonoses [10–13].

Enteroviruses (EVs) are small non-enveloped viruses with positive single strand RNA genomes that belong to the *Picornaviridae* family. Their genome, about 7.5 kb in length, encodes four structural proteins (VP1-VP4) and seven non-structural proteins (2A to 2C and 3A to 3D). EVs consist of more than 300 serotypes and, based on the VP1 sequence, are classified into nine species *EV-A* to *EV-H* and *EV-J* [14]. EVs infecting humans belong to species *EV-A* to *EV-D*, formerly termed *Human Enterovirus A-D*, while the other species contain viruses infecting bovines (*EV-E* and *EV-F*), porcines (*EV-G*) and monkeys (*EV-H* and *EV-J*) [14]. EV-C is associated with various human illnesses, such as the common cold, acute hemorrhagic conjunctivitis, aseptic meningitis and acute flaccid paralysis (AFP). Human EV A-D have been detected in non-human primates, captive or wild Old World monkeys (such as *Macaca sp*, *Cercopithecus sp*, *Cercocebus* and *Papio sp*) and wild African great apes (*Gorilla gorilla* and *Pan troglodytes*) [8, 15–17]. Importantly, both known and novel types of human enteroviruses could be found in primates, indicating common cross-species transmission [18].

In mid-October 2010, a poliomyelitis outbreak occurred in the human population of Point-Noire and surrounded areas and villages in the Republic of Congo (RC). The causative agent of this outbreak was identified as the type 1 wild poliovirus (WPV1), while a novel enterovirus 105 (EV-C105) was detected in one patient. Both PV1 and EV-C105 are members of the *EV-C* species [19, 20]. In November 2010, one month after the start of this outbreak, a chimpanzee (*Pan troglodytes troglodytes*) from the Tchimpounga Chimpanzee Rehabilitation Center developed a syndrome compatible with AFP. The main objective of this study was to identify the pathogen responsible of this syndrome. We identified EV-C99 as a possible causative agent of this chimpanzee’s illness. This is the first identification and characterization of non-polio *EV-C* in a chimpanzee displaying AFP.

## Materials and Methods

### Case report and specimen collection

In November 2010, in the Tchimpounga Chimpanzee Rehabilitation Center located 50 km from Pointe-Noire, a juvenile female chimpanzee presented signs of AFP. This chimpanzee first developed a 38.8°C fever and four days after, she lost the ability of her lower limbs and became quadriplegic. The first clinical examination revealed absence of the osteotendinous reflex, a myasthenia and myalgia after flexion of the lower limbs, and dysphonia. This chimpanzee, a member of a group of twelve individuals, was the only one showing these symptoms of AFP. The other examination based on cerebral scan and x-ray photography did not permit to identify the cause of the disease. After the disease onset, the chimpanzee received treatments of B vitamin and glucose, survived, but did not recover the use of lower limbs, which is a typical sign of AFP caused by poliovirus in humans.

In order to identify the pathogen responsible for AFP, a specimen of feces was collected from this individual (named IJC04). The sample was subsequently stored at -80°C before being sent to the International Centre for Medical Research (CIRMF, Franceville, Gabon) for diagnostic investigations.

### Ethics

The permission to take sample was approved by the Tchimpounga Chimpanzee Rehabilitation Center and the Ministry of Health of Republic of Congo. The fecal sample was collected using a non-invasive method [11].

### EV detection

RNA was extracted from the fecal sample using EZ1 Virus Mini kit (Qiagen, Hilden, DE), according to the manufacturer’s recommended procedure. EV detection was performed by amplifying the 5’-untranslated region (5’-UTR) via a one-step real-time reverse transcription (RT)-PCR, as described in Dierssen et al. [21]. The positive sample was amplified using nested RT-PCR targeting the capsid gene VP1 [22]. Positive amplicons were sequenced in both directions (SEQLAB GmbH, DE) to confirm the results and allow for genetic characterization.

### EV type assignment and phylogenetic analysis

The complete capsid gene VP1 sequence obtained in this study was compared to a database of complete sequences of most serotypes available on GenBank, in order to determine whether this sequence was genetically related to a known EV serotype. Multiple alignments were performed using the ClustalW algorithm and phylogenetic trees were constructed by the neighbor-joining method using the Kimura two-parameter model [23], with 10,000 generated trees based on the MEGA program (version 5.2.2) [24].

### EV full-length genome amplification and metavirome analysis

In order to (i) obtain the full-length EV genome and (ii) validate that EV-C99 was the causative agent, the viral metagenome was obtained via next generation sequencing (NGS).

#### Illumina library preparation and sequencing

RNA was re-extracted with QIAmp Viral RNA Mini kit (Qiagen) according to the manufacturer’s instruction and treated with Turbo DNAse (Life Technologies). RNA was reverse transcribed into cDNA with SuperScript III reverse transcriptase using random hexamer primers (Life Technologies). Generated cDNA was then amplified with the Phi29 enzyme, as described previously [25]. A barcoded library was prepared using the Nextera XT kit (Illumina). Amplified DNA was fragmented into 350 bp and adapters were added for multiplexing samples within the same channel. Sequencing was performed with Illumina Hi-seq 2000 sequencer (Illumina) as recommended by the manufacturer.

#### Bioinformatic and recombination event analyses

All reads were filtered according to quality (Phred score >20). Each viral read was selected using viral database and only the region that matched viral genome was considered. In order to obtain the full-length of viral genome, all reads were initially assembled to contigs with ABYSS software [26] with different values of *k*, and contigs were then assembled into a ‘super assembly’ with the CAP3 program [27]. To look at potential recombination events, similarity plot and bootscanning analysis were conducted using Simplot V3.5.1 software [28]. Similarity plot was done using the Kimura substitution model [23] and the bootscanning analysis was carried out using the neighbor-joining method [29] with a sliding window of 400 nt moving in steps of 20 nt.

#### 16S rRNA amplification and pyrosequencing

Given that bacteria such as *Clostridium botulinum* have been known to be responsible of symptoms comparable to AFP, we evaluated the bacterial community present in the fecal sample of the infected chimpanzee by NGS.

#### Ion Torrent library preparation and sequencing

DNA was extracted from frozen fecal samples using the DNA stool Mini kit (Qiagen), following the manufacturer’s instructions. DNA concentrations were measured using QuBit 2.0 Fluorometer kit (Life Technologies). Bacterial 16S ribosomal RNA (rRNA) genes contain nine “hypervariable regions” (named from V1 to V9) that demonstrate considerable sequence diversity among different bacteria. These regions of bacterial 16S rRNA genes were amplified by PCR using two sets of primers V2-4-8 and V3-6,7–9 available in the Ion 16S Metagenomics kit (Life Technologies). The PCR products were purified using SPRI method (Solid Phase Reversible Immobilization) (Agencourt AMPURE XP). Each amplicon was then assessed for fragment size distribution and DNA concentration using a Bioanalyser 2100 (Agilent Technologies, USA). Library preparation followed the Ion Plus Fragment Library (Life Technologies). Products were first end-repaired and purified using SPRI method (Agencourt AMPURE XP). Then, library was bar-coded using the Ion Xpress Barcode Adapter 1–16 kit (Life Technologies) and sample was again purified using SPRI method. Finally, emulsion PCR and enrichment steps were carried out using the Ion PGM (Life Technologies). Sequencing was undertaken using 316 chips and Ion PGMSequencing 400 on the Ion Torrent Personal Genome Machine (PGM).

#### Bioinformatic analyses

All sequences were processed initially using the Ion Torrent platform-specific pipeline software Ion Reporter (Life Technologies).

#### Viral and bacterial phylogenetic abundances

Phylogenetic abundances were calculated using the R software [30]. Reads of each sample were regrouped according to taxa. Box plots were done using the logarithm of percentages. Then, relative abundances were classified from the most to the least abundant taxa.

## Results

### Molecular detection and characterization of EV

The goal of this work was to identify the pathogen responsible for AFP in a sick chimpanzee (IJC04). Since enteroviruses are often responsible for AFP in humans [31] and since an outbreak due to poliovirus occurred a year before in the human population in RC [19], 50 kilometers from the site where AFP was registered in a chimpanzee, we decided to first screen for the poliovirus. A real-time RT-PCR was positive for EV. Then, a nested RT-PCR allowed the acquisition of the VP1 sequence. This sequence was compared to a database of sequences containing almost all EV serotypes available in GenBank confirming that this chimpanzee was infected with an EV.

**Fig 1:**
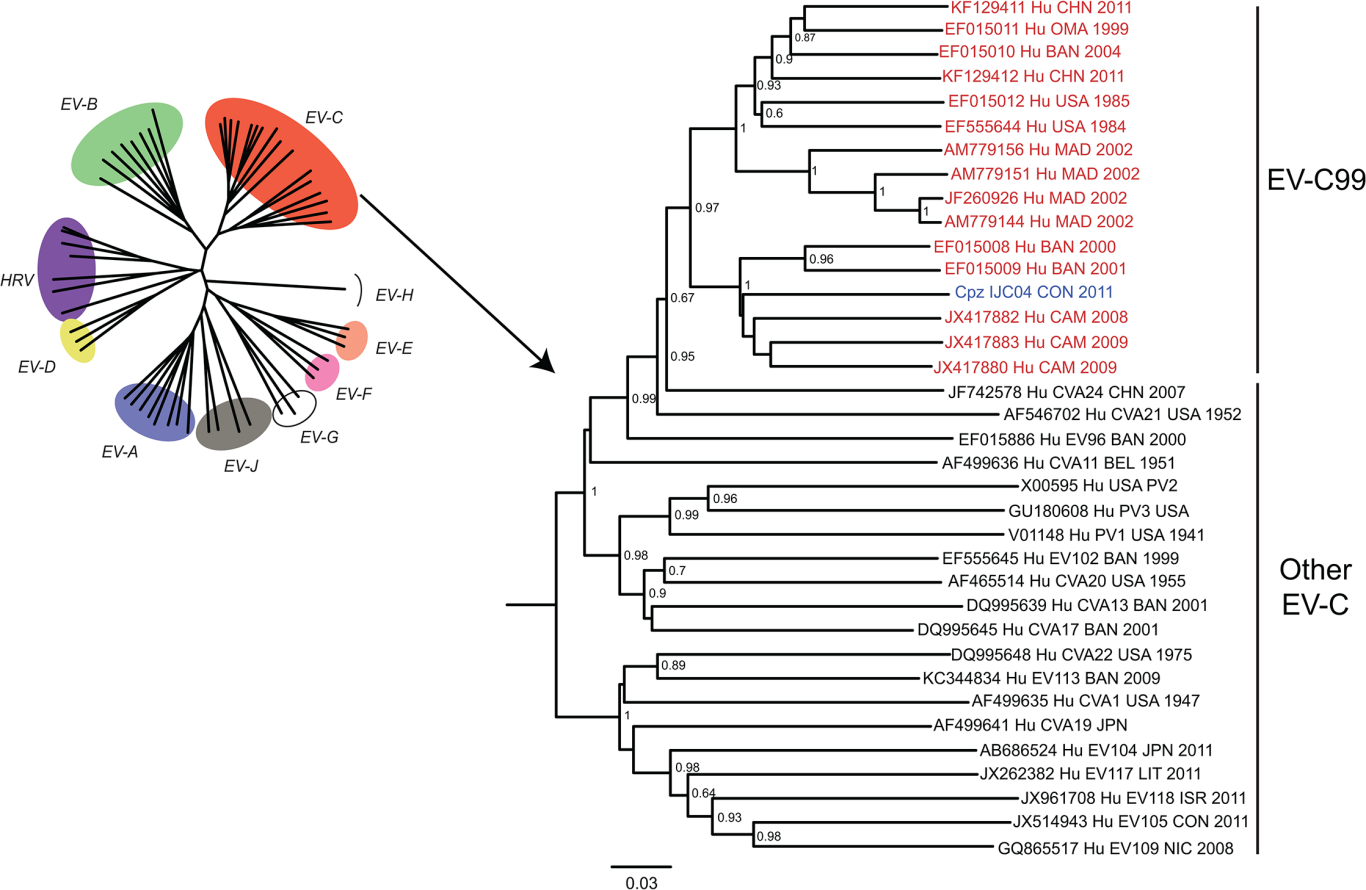
Phylogenetic tree constructed using the entire VP1 sequences (approximately 900 bp) of all EV-*C* serotypes. Study sample is indicated in blue (IJC04) and EV-C99 serotype sequences in red. Accession numbers and information concerning the date of collection and the host species (Hu for human and Cpz for Chimpanzee) are shown. The bootstrap values correspond to 10,000 replicates. Only values above 0.6 are indicated at the nodes. The scale bar represents nucleotide substitutions per site.

Phylogenetic analysis of the complete VP1 sequences of EV species A-D showed that IJC04 isolate was closely related to the *Enterovirus C* species (*EV-C*). IJC04 sequence grouped with EV-C99 serotype sequences and formed a cluster with three Cameroonian EV-C99 strains (JX417880, JX417882 and JX417883) [32] and two EV-C99 strains from Bangladesh BAN00 and BAN01 (EF015008 and EF015009) [33], with a bootstrap value of 100% (Fig 1). The VP1 sequence showed that IJC04 isolate sequence was close to the strains EV-C99 BAN00 (EF015008) and BAN01 (EF015009) isolated in Bangladesh, with 81.1% and 78.2% nucleotide (nt) sequence identities, or 93.7% and 94.7% amino acid (aa) sequence identity respectively and shared 79% to 80.1% nt (93.1% to 94.7% aa) identities with the Cameroonians strains. It is known that EV-C, for example poliovirus, accumulate about 1% of nucleotide substitutions per year [34]. In this context, 20% nt distance would correspond to divergence of human and chimpanzee isolates from a common ancestor about 10 years ago. Bayesian phylogenetic dating was attempted, but did not yield a precise dating of the common ancestor of human and chimpanzee EV-C99 because of the limited sampling of EV-C99 sequences available in Genbank (data not shown).

### EV-C99 full genome analysis

Nearly complete genome was successfully assembled, with 6,648 nucleotides obtained. Despite repetitive attempts, 5'UTR (749 nt) and 3'UTR (72 nt) were not obtained (Table 1). The ORF (Open Reading Frame) encodes for a polyprotein of 2,210 amino acids, and is presumably cleaved in three polyproteins P1 (VP1-VP4, 883 amino acids), P2 (2A-2C, 574 amino acids) and P3 (3A-3D, 753 amino acids) (Table 1).

**Table 1:** Pairwise amino acid sequences identities between IJC04 isolate and other EV-C prototype strains.

		IJC04 Amino acid identity (%)		
Region	Genome location	EV-C99 BAN00 and BAN01	Other EV99	Other EV-C
*VP4*	1–207	98.6–98.6	95.7–98.6	76.8–98.6
*VP2*	208–1020	95.6–96.3	93.4–94.5	70.4–90.0
*VP3*	1021–1740	95.4–96.7	92.9–95.4	70.3–82.9
*VP1*	1741–2649	**93.7–94.7**	86.8–89.1	54.6–81.2
*2A*	2650–3093	88.5–91.9	83.8–88.5	70.3–85.8
*2B*	3094–3384	87.6–91.8	76.8–80.4	56.7–80.4
*2C*	3385–4371	93.3–93.9	86.0–87.2	76.3–86.9
*3A*	4372–4632	88.5–93.1	83.9–88.5	55.2–83.9
*3B*	4633–4698	86.4–90.9	81.8–90.9	72.7–90.9
*3C*	4699–5242	96.7–96.7	91.3–92.9	74.9–90.7
*3D*	5243–6630	94.7–98.3	97.0–99.1	82.6–98.9

**Fig 2:**
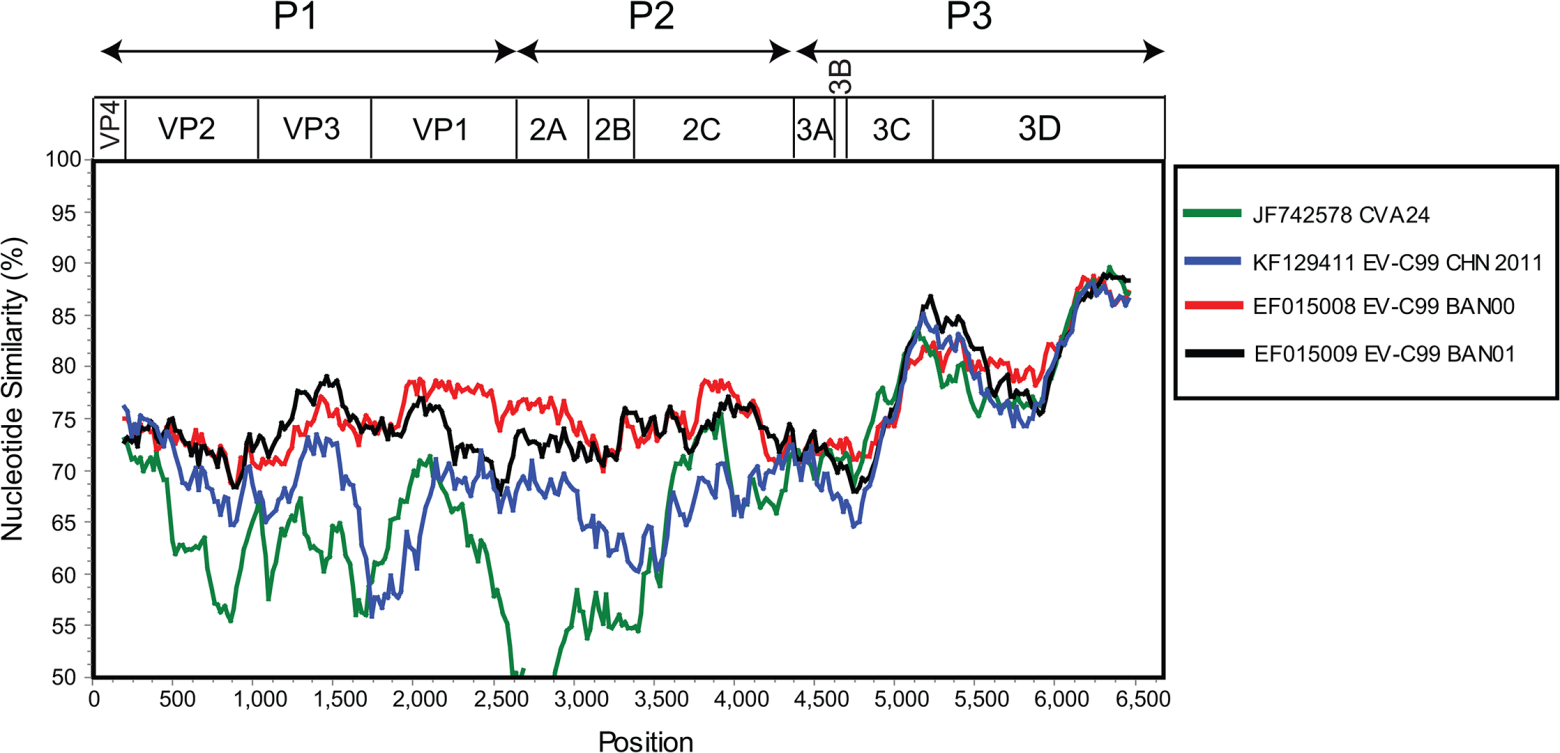
Similarity plot of complete coding region of EV-C99 chimpanzee isolate IJC04 with two EV-C99 sequences from Bangladesh (BAN00 and BAN01), one from China (HT-XEBGH09F), and the next closest serotype Coxsackievirus (CVA24). Similarity plot was performed using a sliding window of 400 nt moving with 20 nt-steps. The coding region structure is provided above the plot.

Similarity plot analysis was used to evaluate nucleotide distance between IJC04, the genetically closest EV-C99 BAN00 and BAN01 strains over the genome (Fig 2). In the P1 and P2 coding regions IJC04 most similar to EV-C99 BAN00 (EF015008) and BAN01 (EF015009). In the P3 there was no single enterovirus sequence clearly most similar to IJC04 (including other Genbank sequences, data not shown).

### NGS overview, viruses and bacteria flora

**Fig 3:**
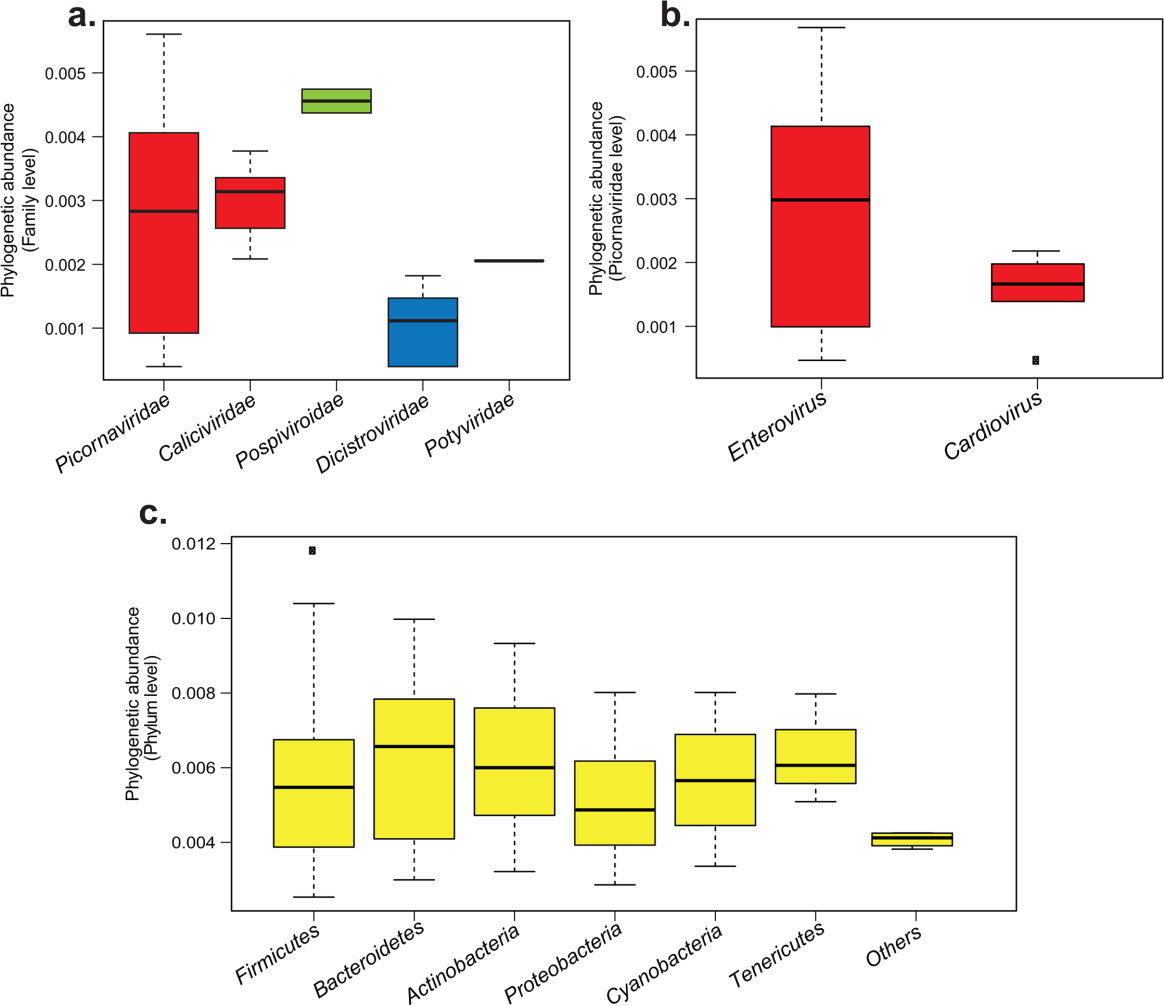
Phylogenetic abundance variations of viral and bacterial reads obtained by NGS in the sample IJC04, a. Viral families phylogenetic abundances, b. Viral genus phylogenetic abundances within the *Picornaviridae* family, c. Bacterial phylogenetic phyla abundances. Box plots are classified from the most to the least abundant group. Boxes represent interquartile range between first and third quartiles and the line inside represents the median. Squares represent outliers beyond the whiskers. The y-axis represents the logarithms of percentage to normalize the values.

A total of 5,049,883 RNA reads were obtained: 11.7% representing 590,472 sequences reads matched RNA viruses and 88.3% of the other reads corresponded to the host genome (the chimpanzee, *Pan troglodytes troglodytes*). Within the RNA viral reads, 98.95% mapped to mammalian viruses (584,283 reads), mainly to *Picornaviridae* (576,942 reads, 98.75%) and the *Caliciviridae* (7,341 reads, 1.25%) families (Fig 3A). Concerning the other reads, 6,189 (1.02%) matched to plant viruses of the *Pospiviroidea* (6006 reads) and the *Potyviridae* families (35 reads) (Fig 3A). Finally, 148 reads (0.02%) matched to insect viruses of the family *Dicistroviridae* (Fig 3A).

Within the *Picornaviridae* family level, reads were distributed among two genera: the *Enterovirus* genus, represented by 576,801 reads (99.6%), and the *Cardiovirus* genus, represented by 141 reads (0.4%) (Fig 3B). Within the *Enterovirus* genus, the EV-*C* species was the most represented in the sample with 541,915 reads (93.95%). The resting reads corresponded to the three other following species *EV-A*, *B* and *D*. Noteworthy, enterovirus 5’ non-translated region (ca. 750 nt, or 10% of the genome) is not species-specific, therefore this finding does not assume presence of additional enteroviruses in the sample.

For the microbiome, out of the total of 589,899 reads obtained, 299,078 (50.7%) reads remained after eliminating unreliable (non-bacterial) or poor quality reads. Sequences were then assembled and a total of 292,948 consensus sequences were obtained. Among these reads, 31 reads did not map to any known bacteria sequences. The rest matched Gram-positive bacteria phyla including *Firmicutes* (232,734 reads, 79.4%), *Actinobacteria* (22,380 reads, 7.9%) and Gram-negative bacteria phyla such as *Bacteroidetes* (27,899 reads, 9.5%), *Proteobacteria* (4,443 reads, 1.51%) of and *Tenericutes* (2,313 reads, 0.79%) (Fig 3C). Finally, 2,397 reads (0.81%) matched photosynthetic bacteria belonging to the phylum of *Cyanobacteria*.

## Discussion

In this study, we report the first identification of an EV-C associated with AFP in a chimpanzee. Enterovirus was detected in fecal sample only from the sick chimpanzee, but not other 11 apes in the group (data not shown). Phylogenetic analysis of the complete capsid gene allowed the identification of the potential causative agent as EV-C99.

In order to rule out other etiologic agents, we investigated the presence of other viral and bacterial agents by mapping metagenomic reads obtained from NGS to available reference genomes. The virus phylogenetic composition of the fecal sample showed that reads corresponded mainly to mammalian viruses belonging to *Caliciviridae* and *Picornaviridae* families. The rest of the reads corresponded to plant and insect viruses, which are very likely derived from chimpanzee's diet and could not be associated to AFP syndrome. Within the *Picornaviridae* family, the *Enterovirus* genus (99%) was the principal representative in terms of reads coverage, followed by the *Cardiovirus* genus with only 0.4% of reads detected. Members of the *Cardiovirus* genus have been associated in non-polio AFP in human populations [35]. However, we could not consider these viruses as the causative agent of the AFP in this chimpanzee. Indeed, a study showed that the viral load was correlated to the number of reads detected by sequencing [36]. The *Caliciviridae* family (1.25%) is composed of viruses mainly responsible for gastroenteritis in human and animal (such as pigs and bats) populations [37–39]. It has to be noticed that in a recent study, we characterized a sapovirus isolated from the same chimpanzee [40]. However, in the current study, this virus was not considered as the causative agent of the AFP observed in this chimpanzee because there are no reports of caliciviruses causing neurological manifestations in humans or primates. Concerning the bacteria flora, mainly commensal bacteria have been identified (95–96%), the resting reads corresponding to telluric bacteria. Indeed, the results showed that the *Firmicutes*, the *Bacteroidetes* and the *Actinobacteria* phyla constituted the vast majority of this chimpanzee’s gut microbiota (Fig 3C). The *Firmicutes* phylum is composed of bacteria with various functions and some are known to be responsible of diseases in human and other vertebrates such as *Clostridium botulinum* (causing a neuroparalytic disease called botulism) [41]. None of the reads obtained in this study matched with *Clostridium botulinum*. Interestingly, the same bacterial flora composition in terms of abundance and diversity is commonly observed in humans [42], as well in gorillas and chimpanzees gut microbiota [43]. In summary, it seems that none of the bacteria detected in this analysis could be responsible of the AFP in this chimpanzee.

As a summary, considering the abundance of EV-C in the fecal sample, neuropathogenic potential of poliovirus and (albeit rarely) of other enteroviruses, EV-C99 is the possible causative agent of the AFP this chimpanzee. However, due to a lack of available serum, detection of antibodies targeting EV-C99 could not performed to better assume this hypothesis. This is the first report of a great ape presenting AFP symptoms associated with a human non-polio EV, member of *EV-C* species. Indeed, historically, the first strain of EV-C99 was identified in Bangladesh in 2000 [33]. Subsequently several other EV-C99 were identified or isolated during poliovirus surveillance from healthy humans or AFP patients in Asia, Europe, North America and Africa [44]. Since this detection, no outbreak has been recorded and no confirmed symptomatic cases were reported. Otherwise, EV-C99 has been identified in one synanthropic rhesus macaque (*Macaca mulatta*) in Bangladesh [18]. In 1964, the Yerkes Regional Primate Research Center (Florida, USA) reported clinical manifestations of AFP in apes caused by poliovirus type 1 [45]. Two years later, another epidemic of paralytic poliomyelitis occurred among chimpanzees of the Gombe National Park (Tanzania), months after a polio outbreak in human population (cited by Dowdle, 1997) [46]. However, laboratory investigations were not performed to determine the exact causative pathogen involved.

The identification of an EV99, commonly circulating in humans, in a captive chimpanzee suggests interspecies transmission from human to chimpanzee. This finding is another example consistent with studies, which demonstrated that non-human primates living in close contact with humans are exposed to the risk of infection with human pathogens [18, 47, 48]. Even though chimpanzees are known to be infected by human and simian EVs, it has to be noticed that this is the first observation of an AFP in a chimpanzee associated to a human EV strain.
